# Supplementary material for: Tandem duplications lead to novel expression patterns through exon shuffling in Drosophila yakuba
Source: PLoS Genet. 2017 May 22;13(5):e1006795. doi: 10.1371/journal.pgen.1006795 (PMC5460883; doi:10.1371/journal.pgen.1006795)
Supplement: S1 Table — (PDF) [file pgen.1006795.s002.pdf]

S1 Table: Genes upregulated using cuffdiff by tissue

Tissue	Duplicates Upregulated	Assayed	Background Upregulated	Assayed	χ^2	P-value
Male Carcass	4	52	1861	13174	0.9697	0.3268
Male Testes	3	52	1375	13174	0.6097	0.4349
Female Carcass	4	52	1733	13174	0.6993	0.4030
Female Ovary	4	52	1343	13174	0.0977	0.7546
