# Supplementary material for: Tandem duplications lead to novel expression patterns through exon shuffling in Drosophila yakuba
Source: PLoS Genet. 2017 May 22;13(5):e1006795. doi: 10.1371/journal.pgen.1006795 (PMC5460883; doi:10.1371/journal.pgen.1006795)
Supplement: S2 Table — (PDF) [file pgen.1006795.s003.pdf]

S2 Table: Whole gene duplications with upregulated expression using Cuffdiff

tissue	gene	strain	Ref FPKM	Sample FPKM	corrected P-value
Male Carcass	2.g417	line9	74.8960	275.4010	0.00102288
	GE11098	line9	36.3276	200.8140	4.0626×10^{-6}
	GE11098	line10	36.3276	132.5500	0.00133
	GE24648	line1	2.7300	9.2186	0.0260
	GE26061	line9	10.9718	28.8023	0.0371
Male Testes	2.g556	line5	0.3080	2.27934	0.0122
	2.g556	line15	0.3080	2.0057	0.0230
	GE11098	line10	620.5090	4025.7000	9.7604×10^{-4}
	GE14157	line13	1.1743	8.2437	0.0030
Female Carcass	GE13159	line19	2.0217	7.96624	0.0031
	GE14157	line2	0.6146	2.8779	0.0046
	GE20775	line8	1.6645	18.4707	2.20709×10^{-5}
	GE26133	line14	22.0725	58.6217	0.0421
Female Ovary	2.g556	line2	0.0686	0.7511	0.0019
	2.g556	line5	0.0686	2.6958	1.13228×10^{-8}
	2.g556	line6	0.0686	1.1139	9.73427×10^{-5}
	2.g556	line9	0.0686	2.0189	3.16552×10^{-7}
	2.g556	line13	0.0686	8.0706	0
	2.g556	line15	0.0686	4.6893	6.89843×10^{-12}
	2.g556	line19	0.0686	1.8320	8.96617×10^{-7}
	GE24648	line1	5.2891	28.7428	1.96519×10^{-7}
	GE26061	line9	8.1007	24.2368	0.0040
	GE24030	line6	2.0279	10.5760	1.17696×10^{-5}
