# Supplementary material for: Tandem duplications lead to novel expression patterns through exon shuffling in Drosophila yakuba
Source: PLoS Genet. 2017 May 22;13(5):e1006795. doi: 10.1371/journal.pgen.1006795 (PMC5460883; doi:10.1371/journal.pgen.1006795)
Supplement: S3 Table — (PDF) [file pgen.1006795.s004.pdf]

S3 Table: Functions of whole gene duplications with upregulated expression

Gene	*D. melanogaster* ortholog	function	
Cuffdiff	*2.g417*	*CG42808*	no function
	GE11098	*Spn38F*	endopepditase. Reproduction. Seminal fluid gene.
	GE24648	*UGt86Di*	glucuronosyltransferase activity, metabolism
	GE26061	*Sodh-2*	Sorbitol dehydrogenase
	2.g556	*CG8834*	coumarate ligase, metabolic process
	GE14157	*Pms2*	mismatch repair, reccombination, MutL alpha complex
	GE13159	*CG13283*	metalloendopeptidase
	GE20775	*Cp16*	chorion protein
	GE26133	*CG14907*	unknown
	GE24030	*CG33099*	gibberellin 20-oxidase activity
HMM	*GE13533*	*γ-trypsin*	endopeptidase
	GE26134	*CG14906*	methyltransferase
	GE13159	*CG13283*	metallo endopeptidase
	2.g417	*CG42808*	unknown
	GE26133	*CG14907*	unknown
