# Supplementary material for: Tandem duplications lead to novel expression patterns through exon shuffling in Drosophila yakuba
Source: PLoS Genet. 2017 May 22;13(5):e1006795. doi: 10.1371/journal.pgen.1006795 (PMC5460883; doi:10.1371/journal.pgen.1006795)
Supplement: S4 Table — (PDF) [file pgen.1006795.s005.pdf]

S4 Table: Genes upregulated using cuffdiff tissue, singleton variants only

Tissue	Duplicates Upregulated	Assayed	Background Upregulated	Assayed	χ^2 (2 *df*)	*P*-value
Male Carcass	2	33	1861	13174	0.8821	0.3476
Male Testes	0	33	1375	13174	2.4248	0.1194
Female Carcass	2	33	1733	13174	0.6826	0.4087
Female Ovary	2	33	1343	13174	0.1844	0.6676
