# Supplementary material for: Tandem duplications lead to novel expression patterns through exon shuffling in Drosophila yakuba
Source: PLoS Genet. 2017 May 22;13(5):e1006795. doi: 10.1371/journal.pgen.1006795 (PMC5460883; doi:10.1371/journal.pgen.1006795)
Supplement: S5 Table — (PDF) [file pgen.1006795.s006.pdf]

S5 Table: SNPs in whole gene duplications with significantly asymmetric expression in male tissues

tissue	gene	strain	chrom	position	Ref DNA	SNP DNA	Ref RNA	SNP RNA	corrected P-value
Male Carcass	0.g329.t1	8	3L	3183795	637	399	2063	2	4.89393946382e-213
	0.g329.t1	8	3L	3184332	651	576	779	2	3.89050343567e-150
	0.g859.t1	8	3L	11082675	165	75	1	12	1.49171249502e-05
	0.g859.t1	8	3L	11082675	165	75	1	12	1.49171249502e-05
	0.g859.t1	8	3L	11082683	171	74	1	13	3.69987951873e-06
	0.g859.t1	8	3L	11082683	171	74	1	13	3.69987951873e-06
	2.g556.t1	6	2R	8629312	58	15	1	10	1.0623042423e-05
	GE10463-PA	13	3R	21435864	55	193	11	4	7.09435847431e-05
	GE10463-PA	13	3R	21435877	66	177	11	3	0.000159872217862
	GE13533-PA	10	2R	9720730	15	28	116	14	1.13741757158e-11
	GE13533-PA	15	2R	9720271	14	22	107	0	8.960918582e-17
	GE13533-PA	15	2R	9720287	14	24	100	0	2.20590551317e-17
	GE13533-PA	15	2R	9720297	13	20	63	0	2.65130711181e-12
	GE13533-PA	15	2R	9720727	16	13	168	2245	1.63605650167e-11
	GE13533-PA	19	2R	9720355	22	26	17	1	0.000234896411228
	GE13533-PA	19	2R	9720857	18	23	30	1	8.68742192434e-07
	GE13533-PA	6	2R	9720839	15	17	51	0	2.77614176204e-09
	GE13533-PA	8	2R	9720271	28	62	40	1	5.85617255782e-14
	GE13533-PA	8	2R	9720287	25	39	36	1	7.34378080343e-10
	GE13533-PA	8	2R	9720839	35	21	39	0	2.38343784016e-06
	GE13533-PA	9	2R	9720610	57	28	96	1162	8.92999655904e-38
	GE13533-PA	9	2R	9720619	67	24	1168	9	3.69329213005e-22
	GE13533-PA	9	2R	9720832	62	40	236	10	7.61814926037e-16
	GE13533-PA	9	2R	9720839	44	37	163	4	3.80443675518e-17
	GE23591-PA	11	3R	25375009	104	42	15	37	1.50368037049e-07
	GE23591-PA	11	3R	25375009	104	42	15	37	1.50368037049e-07
	GE24661-PA	16	3R	10718463	96	55	20	0	0.000492528656681
	GE24661-PA	16	3R	10718959	12	35	11	0	5.93818095445e-06
Male Testes	GE13533-PA	15	2R	9720271	14	22	29	1	3.59877183641e-07
	GE13533-PA	15	2R	9720287	14	24	29	1	1.30865588635e-07
	GE13533-PA	15	2R	9720297	13	20	25	0	4.50871233984e-07
	GE13533-PA	15	2R	9720565	17	9	20	0	0.00298852790899
	GE13533-PA	15	2R	9720727	16	13	3	887	5.81798034916e-24
	GE13533-PA	9	2R	9720610	57	28	8	93	5.47341750466e-18
	GE13533-PA	9	2R	9720619	67	24	93	1	1.09737678153e-07
	GE13533-PA	9	2R	9720832	62	40	23	0	9.23227815148e-05
	GE13533-PA	9	2R	9720839	44	37	16	1	0.00198444795458
	GE19240-PC	2	2L	17480166	102	67	14	0	0.00237723089624
	GE19240-PC	2	2L	17480166	102	67	14	0	0.00237723089624
	GE19240-PC	2	2L	17480167	54	78	15	0	4.7992479603e-06
	GE19240-PC	2	2L	17480167	54	78	15	0	4.7992479603e-06
	GE23591-PA	11	3R	25374738	100	96	16	5	0.0371312257219
	GE23591-PA	11	3R	25374738	100	96	16	5	0.0371312257219
	GE23591-PA	11	3R	25375009	104	42	4	22	1.02571089037e-07
	GE23591-PA	11	3R	25375009	104	42	4	22	1.02571089037e-07
	GE23591-PA	17	3R	25374052	121	62	3	12	0.000612351367959
	GE23591-PA	17	3R	25374052	121	62	3	12	0.000612351367959
	GE23591-PA	17	3R	25374849	94	48	10	19	0.00292326920619
	GE23591-PA	17	3R	25374849	94	48	10	19	0.00292326920619
	GE23591-PA	17	3R	25375009	39	26	6	15	0.0223502005748
	GE23591-PA	17	3R	25375009	39	26	6	15	0.0223502005748
	GE24516-PA	16	3R	12582212	76	36	58	0	4.50226144562e-08
	GE24516-PA	16	3R	12583002	41	59	0	49	2.67572903526e-09
	GE24516-PA	16	3R	12583021	44	33	36	0	2.51789090039e-07
	GE24661-PA	16	3R	10718463	96	55	12	0	0.00880153601503
	GE24661-PA	16	3R	10718791	29	68	0	18	0.0058982953165
