# Supplementary material for: Tandem duplications lead to novel expression patterns through exon shuffling in Drosophila yakuba
Source: PLoS Genet. 2017 May 22;13(5):e1006795. doi: 10.1371/journal.pgen.1006795 (PMC5460883; doi:10.1371/journal.pgen.1006795)
Supplement: S6 Table — (PDF) [file pgen.1006795.s007.pdf]

S6 Table: SNPs in whole gene duplications with significantly asymmetric expression in female tissues

tissue	gene	strain	chrom	position	Ref Genomic	SNP Genomic	Ref RNA-seq	SNP RNA-seq	corrected P-value
Female Carcass	2.g556.t1	9	2R	8628170	52	61	13	0	0.000134635282947
	GE13533-PA	2	2R	9720296	7	21	249	2	1.37883018113e-23
	GE13533-PA	2	2R	9720297	7	22	248	0	7.61164181427e-27
	GE13533-PA	6	2R	9720271	21	11	38	0	5.9627472227e-05
	GE13533-PA	6	2R	9720287	16	10	38	0	3.50671569654e-05
	GE13533-PA	6	2R	9720839	15	17	39	0	5.46168656421e-08
	GE13533-PA	8	2R	9720271	28	62	60	0	1.73436067901e-20
	GE13533-PA	8	2R	9720287	25	39	58	0	4.23964503844e-15
	GE13533-PA	8	2R	9720839	35	21	5647	6	2.64131038422e-39
	GE13533-PA	9	2R	9720610	57	28	67	1214	1.36030183298e-44
	GE13533-PA	9	2R	9720619	67	24	1177	2	4.3505030628e-27
	GE13533-PA	9	2R	9720832	62	40	619	12	1.53640332343e-27
	GE13533-PA	9	2R	9720839	44	37	335	1	4.32719602547e-29
	GE13533-PA	10	2R	9720730	15	28	468	4	3.64633793886e-31
	GE13533-PA	15	2R	9720271	14	22	445	0	6.83413786614e-29
	GE13533-PA	15	2R	9720287	14	24	438	1	1.36160863106e-29
	GE13533-PA	15	2R	9720297	13	20	294	0	1.29111129899e-23
	GE13533-PA	15	2R	9720727	16	13	658	1	8.38049203847e-19
	GE13533-PA	19	2R	9720355	22	26	48	1	1.91720088454e-09
	GE13533-PA	19	2R	9720857	18	23	41	2	1.44452774476e-07
	GE13533-PA	19	2R	9720875	20	38	0	32	4.15947174032e-05
	GE20775-PA	8	3L	3185046	570	372	25	0	7.36488931564e-06
	GE20775-PA	8	3L	3185052	536	357	25	0	7.26234794398e-06
	GE20775-PA	8	3L	3185112	377	517	22	0	8.20383183941e-09
	GE20775-PA	8	3L	3185229	178	162	18	0	2.27731906792e-05
Female Ovary	0.g329.t1	8	3L	3183371	300	246	24	0	1.43200054203e-06
	0.g329.t1	8	3L	3183545	524	329	29	0	1.44529786542e-06
	GE19240-PC	2	2L	17480167	54	78	14	0	1.0224484785e-05
	GE19240-PC	2	2L	17480167	54	78	14	0	1.0224484785e-05
	GE21202-PA	9	3L	1897080	96	42	47	0	1.04727614702e-06
	GE24516-PA	16	3R	12582212	76	36	247	4	1.43363038229e-16
	GE24516-PA	16	3R	12583002	41	59	31	158	7.49321729398e-06
	GE24516-PA	16	3R	12583021	44	33	155	24	9.30200258675e-07
