# Supplementary material for: Tandem duplications lead to novel expression patterns through exon shuffling in Drosophila yakuba
Source: PLoS Genet. 2017 May 22;13(5):e1006795. doi: 10.1371/journal.pgen.1006795 (PMC5460883; doi:10.1371/journal.pgen.1006795)
Supplement: S7 Table — (PDF) [file pgen.1006795.s008.pdf]

S7 Table: Upregulated sites genomewide

Chromosome	Upregulated	All	Proportion
2L	17866800	22324452	0.0534
2R	19202652	21139217	0.0606
3L	18281473	24197627	0.0504
3R	22455173	28832112	0.0519
X	15544647	21770863	0.0476
All	93350745	118264271	0.0526
