# Supplementary material for: Tandem duplications lead to novel expression patterns through exon shuffling in Drosophila yakuba
Source: PLoS Genet. 2017 May 22;13(5):e1006795. doi: 10.1371/journal.pgen.1006795 (PMC5460883; doi:10.1371/journal.pgen.1006795)
Supplement: S8 Table — (PDF) [file pgen.1006795.s009.pdf]

S8 Table: Upregulated genes

Chimeras	Tissue	$\geq$ 50 bp Upregulated	Total
	Female Carcass	39	76
	Female Ovary	40	76
	Male Carcass	41	76
	Male Testes	44	76
	All	55	76
Whole Gene	Tissue	$\geq$ 50 bp Upregulated	Total
	Female Carcass	17	66
	Female Ovary	18	66
	Male Carcass	20	66
	Male Testes	18	66
	All	36	66
