# Supplementary material for: Tandem duplications lead to novel expression patterns through exon shuffling in Drosophila yakuba
Source: PLoS Genet. 2017 May 22;13(5):e1006795. doi: 10.1371/journal.pgen.1006795 (PMC5460883; doi:10.1371/journal.pgen.1006795)
Supplement: S9 Table — (PDF) [file pgen.1006795.s010.pdf]

S9 Table: Length of 'de novo' gene segments

tissue	chromosome	start	stop	strain	size (bp)
Male Carcass	2L	4764717	4771771	1	201
	2L	7100699	7103913	6	212
	2L	7043543	7048586	5	217
	2L	7043543	7048586	9	224
	2L	22076307	22081156	13	237
	2L	22076307	22081156	5	246
	2L	22217615	22221738	17	248
	2L	22076307	22081156	15	254
	3L	7643207	7647178	6	256
	2L	7043543	7048586	10	256
	2R	1296122	1299376	19	380
	2R	1298866	1302456	19	380
	2L	22076307	22081156	14	384
	3R	14703209	14705506	11	754
	2R	550564	555698	13	1364
Male Testes	2R	8628288	8637097	5	202
	2L	14844348	14850368	2	205
	2L	19481376	19484185	11	214
	2L	21809552	21814176	5	227
	3R	15663794	15666868	8	234
	2L	21860804	21864242	19	245
	X	8626278	8645156	12	256
	2R	1296122	1299376	13	278
	2R	1298866	1302456	13	278
	2L	22076307	22081156	5	292
	2R	1296122	1299376	19	303
	2R	1298866	1302456	9	304
	3R	28773101	28773775	8	306
	2L	7043543	7048586	9	326
	2R	12531901	12536511	10	327
	2L	1858014	1866626	19	353
	2L	4764717	4771771	1	353
	2R	1298866	1302456	19	355
	2L	22229672	22240590	12	374
	2L	22076307	22081156	13	380
	2R	261487	266019	2	381
	2R	13593056	13597666	11	387
	3L	15707277	15731097	6	412
	2L	5056039	5058911	5	428
	2R	15243572	15249038	6	481
	3R	7559447	7567609	6	569
	3R	14703209	14705506	11	575
	2L	22076307	22081156	14	594
	2L	22229672	22240590	13	846
Female Carcass	3L	7643207	7647178	6	204
	2L	22076307	22081156	15	227
	2L	22076307	22081156	13	228
	2R	1298866	1302456	13	231
	2L	6538411	6540646	1	258
	2R	1296122	1299376	13	353
	3R	14703209	14705506	11	770
	2R	550564	555698	13	1056
Female Ovary	X	21252863	21277771	14	343
	2R	1340493	1343865	6	686
