# Supplementary material for: Tandem duplications lead to novel expression patterns through exon shuffling in Drosophila yakuba
Source: PLoS Genet. 2017 May 22;13(5):e1006795. doi: 10.1371/journal.pgen.1006795 (PMC5460883; doi:10.1371/journal.pgen.1006795)
Supplement: S10 Table — (PDF) [file pgen.1006795.s011.pdf]

S10 Table: FPKM for recruited non-coding parental genes

Gene	Female Carcass	Female Ovary	Male Carcass	Male Testes
GE19344	1.11753	4.32018	0.956197	1.4404
GE20665	28.1935	19.4317	29.7131	17.7321
2.g418	8.6822	0.0	3.83027	3.80075
GE14641	0.00866161	0.0	3.06248	51.3189
GE14103	36.4129	135.039	28.3912	86.1859
3.g1278	9.08642	10.7779	10.0527	11.0089
GE20792	10.8708	21.321	8.42913	5.57496
GE17340	0.77299	20.1854	3.78907	79.9769
GE22019	68.3313	9.05024	89.1704	43.2689
2.g418	8.6822	0.0	3.83027	3.80075
GE26314	5.25971	12.9278	3.99471	8.6008
4.g321	0.935342	0.405618	0.908753	0.551468
1.g396	0.548732	0.0167492	1.65429	26.874
GE22133	14.7227	83.3471	15.9165	23.4822
GE18873	7.2591	55.5697	5.87663	23.0839
GE18174	58.0966	34.1165	55.9907	31.9375
GE19410	13.1419	47.2782	13.6642	88.2691
GE22569	0.0237261	0.0	0.0238531	0.419282
GE15832	0.0205719	0.150601	0.0233789	0.158375
2.g1622	0.484331	0.0	0.083226	0.213026
0.g951	0.125324	0.00351728	0.258543	0.0405134
GE21054	0.776906	1.85926	0.526995	1.64772
1.g5	2.82885	0.484007	3.67932	3.00005
GE16826	8.67266	38.7514	8.17896	8.1714
GE13040	0.0291575	0.0112303	0.13833	0.716646
GE13038	1.44773	0.0	5.34928	0.369484
GE21286	7.33772	35.3616	5.99842	25.6466
GE12967	1.9533	5.76889	1.81867	1.42007
1.g1354	0.0419969	0.0	0.111257	0.092872
GE16584	2.03507	16.8762	2.12453	5.10268
GE26259	40.6827	0.128916	15.1555	10.2535
GE12967	1.9533	5.76889	1.81867	1.42007
GE16953	0.120014	0.537476	0.120596	0.0690305
2.g361	0.00773397	0.0	0.0	0.0192081
GE26071	8.55841	0.68632	4.21104	2.41655
GE16978	3.92049	17.4673	3.13624	4.64776
GE13160	0.561831	0.215925	0.972459	11.6932
GE15086	2.49297	0.0892037	7.21166	1.44863
2.g1732	0.474107	0.0	1.4353	0.164268
GE17162	3.17218	16.647	3.65224	1.82999
GE10771	0.0902181	0.011496	0.110188	0.0291091
3.g15	0.0	0.510221	0.0	0.0
GE12967	1.9533	5.76889	1.81867	1.42007
