# Supplementary material for: Tandem duplications lead to novel expression patterns through exon shuffling in Drosophila yakuba
Source: PLoS Genet. 2017 May 22;13(5):e1006795. doi: 10.1371/journal.pgen.1006795 (PMC5460883; doi:10.1371/journal.pgen.1006795)
Supplement: S11 Table — (PDF) [file pgen.1006795.s012.pdf]

S11 Table: Ancestral Expression Patterns

Tissue	Dup Mean FPKM	All Mean FPKM	Dup Median FPKM	All Median FPKM	Wilcox W	P-value
Ovary	23.12815	16.65176	0.5913	0.3053	8254952	3.291×10^{-4}
Female Carcass	19.0621	16.8729	2.6573	1.3851	8884288	2.282×10^{-16}
Testes	17.78303	15.1603	3.3762	1.9954	8743698	7.368×10^{-13}
Male Carcass	20.34798	17.2835	9040304	3.3519	1.9687	2.2×10^{-16}
