# Supplementary material for: Tandem duplications lead to novel expression patterns through exon shuffling in Drosophila yakuba
Source: PLoS Genet. 2017 May 22;13(5):e1006795. doi: 10.1371/journal.pgen.1006795 (PMC5460883; doi:10.1371/journal.pgen.1006795)
Supplement: S12 Table — (PDF) [file pgen.1006795.s013.pdf]

S12 Table: Ancestral Expression Patterns for Variants in $\leq \frac{2}{20}$ Strains

Tissue	Dup Mean FPKM	All Mean FPKM	Wilcox W	P-value
Ovary	24.170	16.650	7207434	4.148×10^{-5}
Female Carcass	18.910	16.879	7686539	2.694×10^{-15}
Testes	17.300	15.160	7588718	1.045×10^{-12}
Male Carcass	20.912	17.284	7844719	2.2×10^{-16}
