# Supplementary material for: Tandem duplications lead to novel expression patterns through exon shuffling in Drosophila yakuba
Source: PLoS Genet. 2017 May 22;13(5):e1006795. doi: 10.1371/journal.pgen.1006795 (PMC5460883; doi:10.1371/journal.pgen.1006795)
Supplement: S13 Table — (PDF) [file pgen.1006795.s014.pdf]

S13 Table: Sample strains surveyed

Stock Number	Strain
14021-0261.01	Reference
14021-0261.39	CY04B
14021-0261.40	CY08A
14021-0261.41	CY17C
14021-0261.42	CY20A
14021-0261.43	CY21B3
14021-0261.44	CY22B
14021-0261.47	NY48
14021-0261.48	NY56
14021-0261.49	NY62
14021-0261.50	NY65
14021-0261.51	NY66-2
14021-0261.52	NY73
14021-0261.53	NY81
14021-0261.54	NY85
N/A	CY28A
