# Supplementary material for: Tandem duplications lead to novel expression patterns through exon shuffling in Drosophila yakuba
Source: PLoS Genet. 2017 May 22;13(5):e1006795. doi: 10.1371/journal.pgen.1006795 (PMC5460883; doi:10.1371/journal.pgen.1006795)
Supplement: S14 Table — (PDF) [file pgen.1006795.s015.pdf]

S14 Table: Upregulated genes using Baum-Welch transition probabilities

Chimeras	Tissue	Upregulated	Total
	Female Carcass	5	76
	Female Ovary	10	76
	Male Carcass	10	76
	Male Testes	9	76
	All	22	76
Whole Gene	Tissue	Upregulated	Total
	Female Carcass	3	66
	Female Ovary	2	66
	Male Carcass	1	66
	Male Testes	1	66
	All	5	66
