# Supplementary material for: Tandem duplications lead to novel expression patterns through exon shuffling in Drosophila yakuba
Source: PLoS Genet. 2017 May 22;13(5):e1006795. doi: 10.1371/journal.pgen.1006795 (PMC5460883; doi:10.1371/journal.pgen.1006795)
Supplement: S2 Fig — Chimeric genes are more likely to result in high mean fold change than unmutated counterparts in all tissues. Whole gene duplicates create multifold expression changes more rarely. (PDF) [file pgen.1006795.s017.pdf]

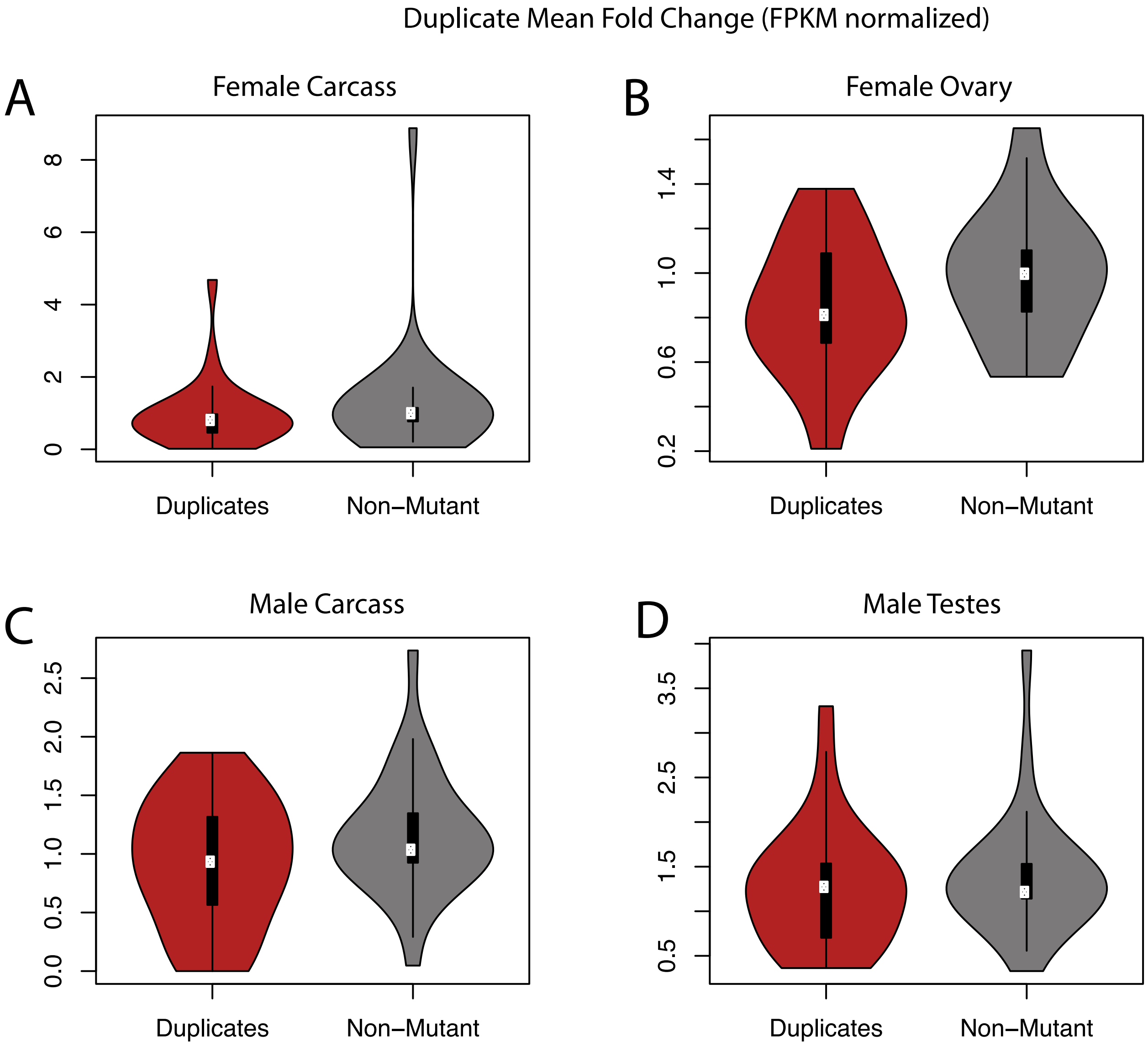

S2 Figure: Mean fold change using FPKM normalized data for chimeric genes in sample strains vs. reference for strains containing chimeras or whole gene duplicates (red) and unmutated sample strains for the same regions (grey). Chimeric genes are more likely to result in high mean fold change than unmutated counterparts in all tissues. Whole gene duplicates create multifold expression changes more rarely.
