# Supplementary material for: Tandem duplications lead to novel expression patterns through exon shuffling in Drosophila yakuba
Source: PLoS Genet. 2017 May 22;13(5):e1006795. doi: 10.1371/journal.pgen.1006795 (PMC5460883; doi:10.1371/journal.pgen.1006795)
Supplement: S3 Fig — The tandem duplication also captures the entire gene sequence of GE26134, as well as portions of GE26132 and GE24588. The duplicate exhibits greater than two-fold expression of GE26133 in the sample strain containing the duplication. It is unclear whether the expression change is a direct consequence of duplication, secondary mutation, environmental effects, or other stochastic variation in expression. (PDF) [file pgen.1006795.s018.pdf]

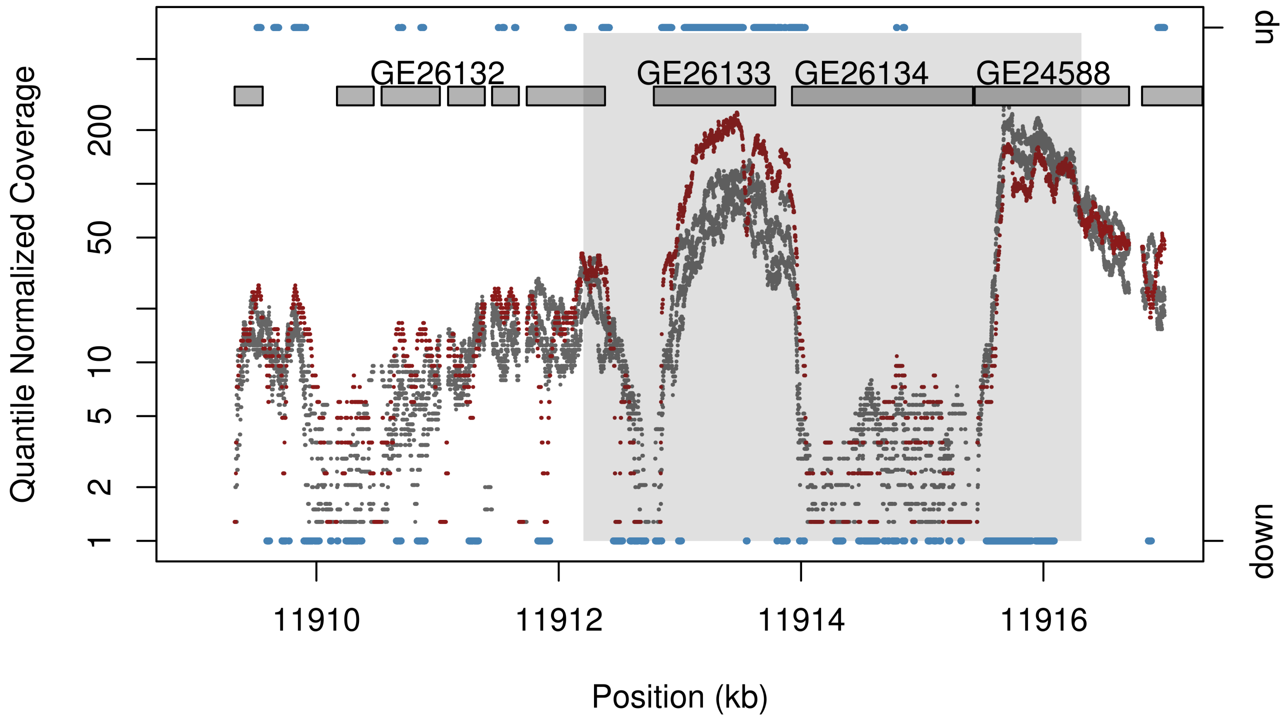


S3 Figure: Expression change in a sample strain containing a whole gene duplication of *GE26133* (reference FPKM=22.0725, sample FPKM=58.6217, uncorrected $P = 0.00263417$, corrected $P = 0.0420917$). The tandem duplication also captures the entire gene sequence of *GE26134*, as well as portions of *GE26132* and *GE24588*. The duplicate exhibits greater than two-fold expression of *GE26133* in the sample strain containing the duplication. It is unclear whether the expression change is a direct consequence of duplication, secondary mutation, environmental effects, or other stochastic variation in expression.
