# Supplementary material for: Tandem duplications lead to novel expression patterns through exon shuffling in Drosophila yakuba
Source: PLoS Genet. 2017 May 22;13(5):e1006795. doi: 10.1371/journal.pgen.1006795 (PMC5460883; doi:10.1371/journal.pgen.1006795)
Supplement: S4 Fig — Quantile normalized coverage in a single sample vs. the mean of quantile normalized coverage in the reference for sites with upregulated sequence are plotted in red, while that of down regulated sequence is shown in blue for 500,000 bp beginning at 6.5 Mb on chromosome 3L for sites with quantile normalized coverage ≤ 500. Sites with no expression change identified using the HMM are not shown. The case of equal expression is shown with the black solid line, while two-fold coverage increase in the sample are indicated with the dashed line. Even modest increases in expression can be identified with the HMM, suggesting that its ability to detect site level differences in high coverage RNA-seq data is high. (PDF) [file pgen.1006795.s019.pdf]

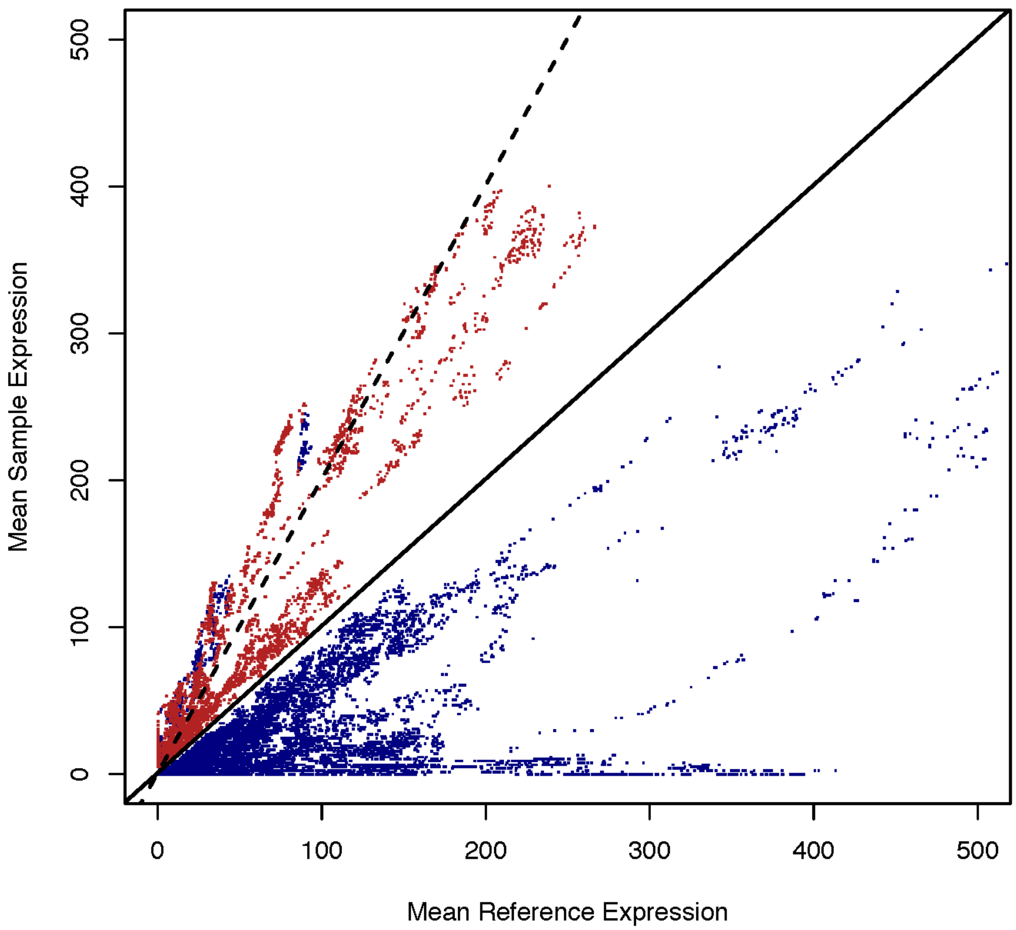

S4 Figure: HMM Performance in quantile normalized coverage data. Quantile normalized coverage in a single sample vs. the mean of quantile normalized coverage in the reference for sites with upregulated sequence are plotted in red, while that of down regulated sequence is shown in blue for 500,000 bp beginning at 6.5 Mb on chromosome 3L for sites with quantile normalized coverage ≤ 500. Sites with no expression change identified using the HMM are not shown. The case of equal expression is shown with the black solid line, while two-fold coverage increase in the sample are indicated with the dashed line. Even modest increases in expression can be identified with the HMM, suggesting that its ability to detect site level differences in high coverage RNA-seq data is high.
