# Supplementary material for: Tandem duplications lead to novel expression patterns through exon shuffling in Drosophila yakuba
Source: PLoS Genet. 2017 May 22;13(5):e1006795. doi: 10.1371/journal.pgen.1006795 (PMC5460883; doi:10.1371/journal.pgen.1006795)
Supplement: S5 Fig — The deleted segment is supported by a decrease in genome coverage as well as 104 long-spanning Illumina sequencing reads. Coverage increases two-fold to three-fold in the duplicated segment, and is not supportive of higher level copy number that might explain the increase in expression as defined by RNA-seq data. HMM output for the region with increased expression in RNA-seq data is shown in blue, for comparison. The region the gene segment with the expression change corresponds well with the region displaying elevated genomic sequencing coverage given the structure of ancestral gene models (see Fig 3). (PDF) [file pgen.1006795.s020.pdf]

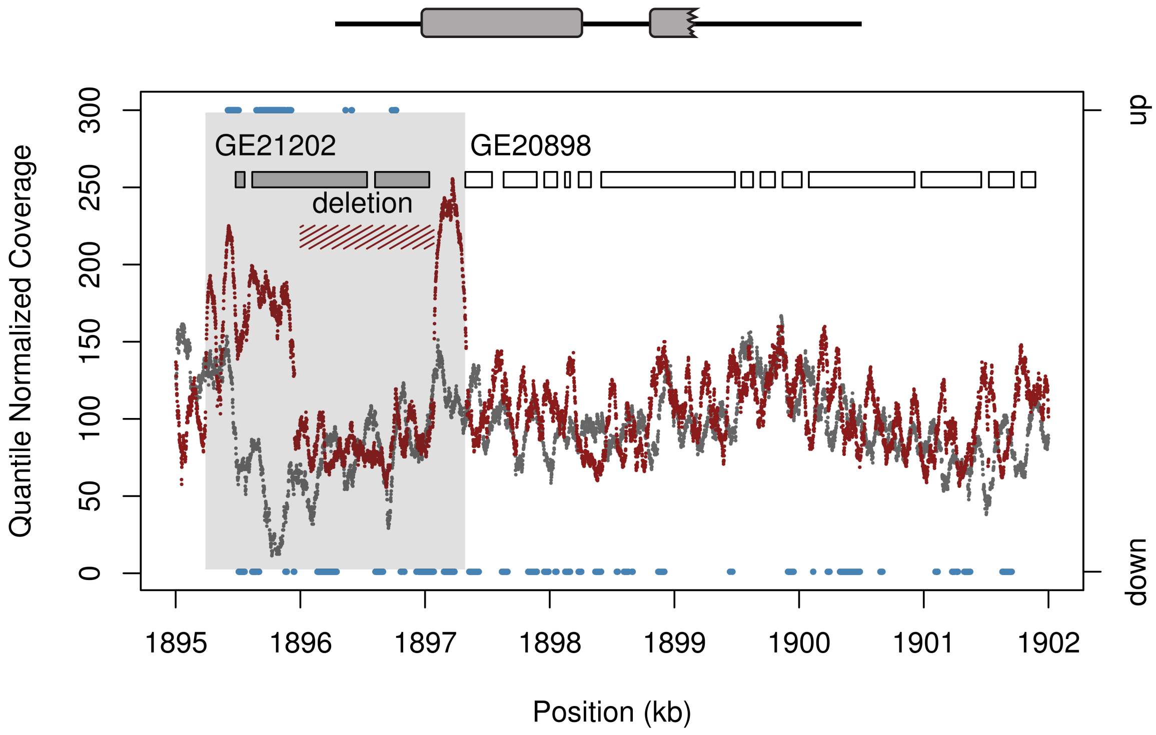

S5 Figure: Genomic DNA sequencing coverage in the sample (red) and resequenced reference (grey) [17] and RNA-seq HMM Expression output for a region experiencing a secondary deletion after duplication. The deleted segment is supported by a decrease in genome coverage as well as 104 long-spanning Illumina sequencing reads. Coverage increases two-fold to three-fold in the duplicated segment, and is not supportive of higher level copy number that might explain the increase in expression as defined by RNA-seq data. HMM output for the region with increased expression in RNA-seq data is shown in blue, for comparison. The region the gene segment with the expression change corresponds well with the region displaying elevated genomic sequencing coverage given the structure of ancestral gene models (see Figure 3).
