# Supplementary material for: Tandem duplications lead to novel expression patterns through exon shuffling in Drosophila yakuba
Source: PLoS Genet. 2017 May 22;13(5):e1006795. doi: 10.1371/journal.pgen.1006795 (PMC5460883; doi:10.1371/journal.pgen.1006795)
Supplement: S6 Fig — A) A tandem duplication captures the 5′ segment of GE18453 and the 3′ segment of GE18451. The tandem duplication unites these gene segments to form a novel open reading frame distinct from the parental genes. Shuffling of regulatory elements in the 5′ and 3′ ends results in a new regulatory profile for the chimera. The tandem duplication also copies the full gene sequence of GE18452. B) A tandem duplication captures the 5′ end of GE24349, placing it next to previously untranscribed sequence. The promoter and UTR of GE24349 drives expression of a previously untranscribed region. (PDF) [file pgen.1006795.s021.pdf]

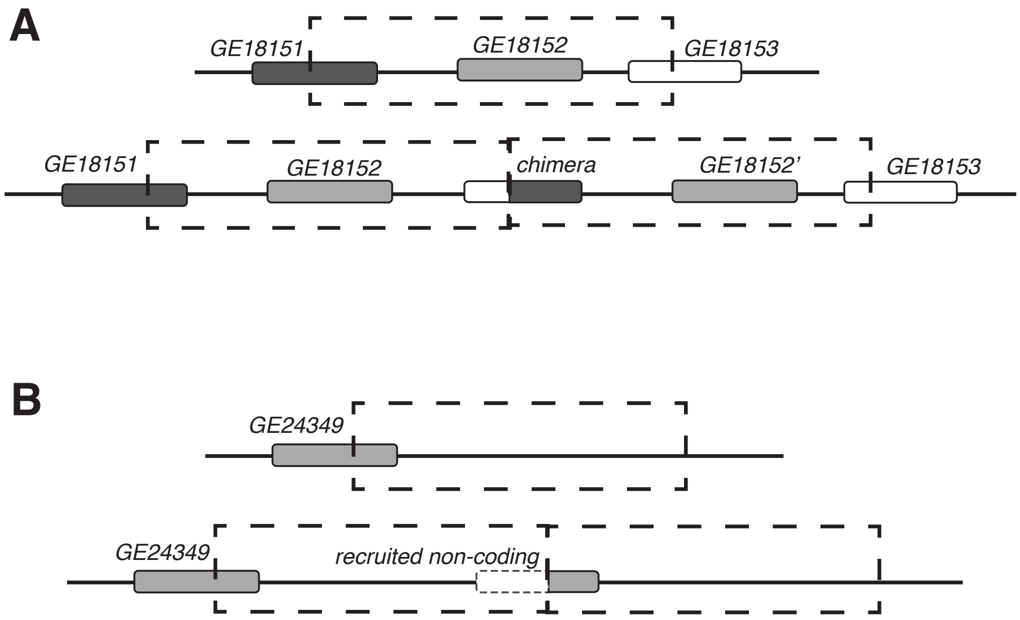

S6 Figure: Formation of alternative gene structures through tandem duplications. A) A tandem duplication captures the 5′ segment of *GE18453* and the 3′ segment of *GE18451*. The tandem duplication unites these gene segments to form a novel open reading frame distinct from the parental genes. Shuffling of regulatory elements in the 5′ and 3′ ends results in a new regulatory profile for the chimera. The tandem duplication also copies the full gene sequence of *GE18452*. B) A tandem duplication captures the 5′ end of *GE24349*, placing it next to previously untranscribed sequence. The promoter and UTR of *GE24349* drives expression of a previously untranscribed region.
