# Supplementary material for: Tandem duplications lead to novel expression patterns through exon shuffling in Drosophila yakuba
Source: PLoS Genet. 2017 May 22;13(5):e1006795. doi: 10.1371/journal.pgen.1006795 (PMC5460883; doi:10.1371/journal.pgen.1006795)
Supplement: S7 Fig — RNA-seq data shows differentiation between intron and exon sequence and spans the entire length of the the transcript. (PDF) [file pgen.1006795.s022.pdf]

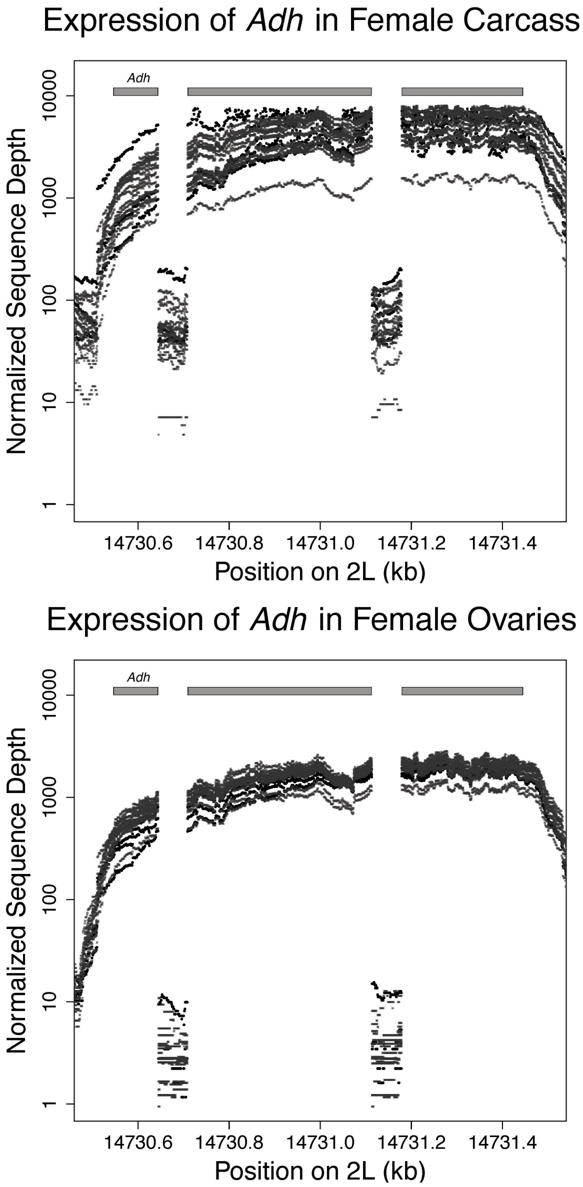

S7 Figure: Normalized coverage in RNA-seq Data for *Adh* in 15 sample strains and 3 replicates of the reference. RNA-seq data shows differentiation between intron and exon sequence and spans the entire length of the the transcript.
