# Supplementary material for: Tandem duplications lead to novel expression patterns through exon shuffling in Drosophila yakuba
Source: PLoS Genet. 2017 May 22;13(5):e1006795. doi: 10.1371/journal.pgen.1006795 (PMC5460883; doi:10.1371/journal.pgen.1006795)
Supplement: S9 Fig — Coverage varies across strains, but is generally high with thousands of reads for the most highly expressed genes. To reduce variability in coverage and generate more robust differential expression calls, we quantile normalized coverage inputs for the HMM. (PDF) [file pgen.1006795.s024.pdf]

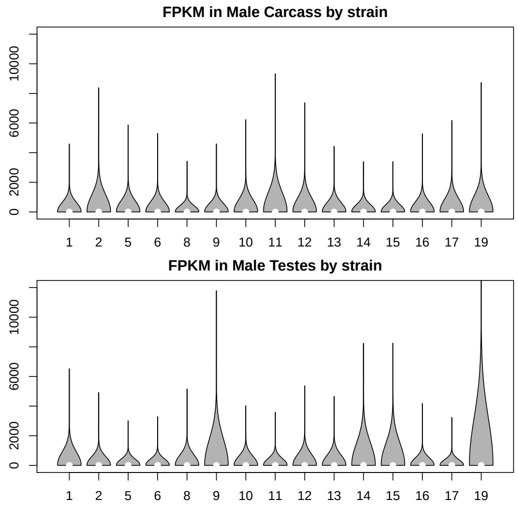

S9 Figure: FPKM values in RNA-seq data in male tissues for 15 sample strains. Coverage varies across strains, but is generally high with thousands of reads for the most highly expressed genes. To reduce variability in coverage and generate more robust differential expression calls, we quantile normalized coverage inputs for the HMM.
